# CYFIP2 serves as a prognostic biomarker and correlates with tumor immune microenvironment in human cancers

**DOI:** 10.1186/s40001-023-01366-2

**Published:** 2023-09-21

**Authors:** Qiliang Peng, Bixin Ren, Kedao Xin, Weihui Liu, Md Shahin Alam, Yinyin Yang, Xuhao Gu, Yaqun Zhu, Ye Tian

**Affiliations:** 1 Department of Radiotherapy & Oncology, The Second Affiliated Hospital of Soochow University, San Xiang Road No. 1055, Suzhou, 215004 Jiangsu China; 2 Institute of Radiotherapy & Oncology, Soochow University, Suzhou, China; 3 State Key Laboratory of Radiation Medicine and Protection, Soochow University, Suzhou, China; 4 Department of Respiratory and Critical Care Medicine, The Second Affiliated Hospital of Soochow University, Suzhou, China; 5 Department of Radiation Oncology, Suzhou Hospital, Affiliated Hospital of Medical School, Nanjing University, Suzhou, China; 6 Department of Oncology, Dazhou Central Hospital, Dazhou, China; 7 Laboratory of Molecular Neuropathology, Department of Pharmacology, Jiangsu Key Laboratory of Neuropsychiatric Diseases and College of Pharmaceutical Sciences, Soochow University, Suzhou, China

**Keywords:** CYFIP2, Immuno-oncology, Biomarker, Prognosis

## Abstract

**Background:**

The mechanisms whereby CYFIP2 acts in tumor development and drives immune infiltration have been poorly explored. Thus, this study aimed to identifying the role of CYFIP2 in tumors and immune response.

**Methods:**

In this study, we first explored expression patterns, diagnostic role and prognostic value of CYFIP2 in cancers, particularly in lung adenocarcinoma (LUAD). Then, we performed functional enrichment, genetic alterations, DNA methylation analysis, and immune cell infiltration analysis of CYFIP2 to uncover its potential mechanisms involved in immune microenvironment.

**Results:**

We found that CYFIP2 significantly differentially expressed in different tumors including LUAD compared with normal tissues. Furthermore, CYFIP2 was found to be significantly correlated with clinical parameters in LUAD. According to the diagnostic and survival analysis, CYFIP2 may be employed as a potential diagnostic and prognostic biomarker. Moreover, genetic alterations revealed that mutation of CYFIP2 was the main types of alterations in different cancers. DNA methylation analysis indicated that CYFIP2 mRNA expression correlated with hypomethylation. Afterwards, functional enrichment analysis uncovered that CYFIP2 was involved in tumor-associated and immune-related pathways. Immune infiltration analysis indicated that CYFIP2 was significantly correlated with immune cells infiltration. In particular, CYFIP2 was strongly linked with immune microenvironment scores. Additionally, CYFIP2 exhibited a significant relationship with immune regulators and immune-related genes including chemokines, chemokines receptors, and MHC genes.

**Conclusion:**

Our results suggested that CYFIP2 may serve as a prognostic cancer biomarker for determining prognosis and might be a promising therapeutic strategy for tumor immunotherapy.

## Introduction

The occurrence and development of malignancies may be affected by many factors, such as genetics, living environment and habits. At present, the efficacy of tumor treatment is still far from satisfactory, probably because the occurrence of tumor is linked to genetic variants [1]. In-depth knowledge of the full range of functions of a gene is meaningful to help us better understand the mechanism of tumorigenesis and promote personalized treatment [2]. With the rapid development of basic research and clinical medicine, people’s understanding of the etiology and pathophysiological processes of diseases has been deepened, and more and more biomarkers involved in tumorigenesis, development and influencing prognosis have been discovered one after another, and play an important role in early diagnosis of tumors, evaluation of therapeutic efficacy and prediction of prognosis [3]. Sensitive and specific biomarkers are especially necessary in clinical precision medicine [4]. Biomarkers can also be used as potential targets for drug design. The discovery of powerful biomarkers and new therapeutic targets is critical to the development of the next generation of precision medicine. During past years, the improvement of high-throughput sequencing based cancer atlas initiatives and omics technologies has brought cancer research into a new era [5]. Computational or bioinformatics approaches have made it more efficient to investigate the ability and function of target molecules as well as their interactions, thereby contributing to a comprehensive understanding of cancer initiation and progression [6].

Lung adenocarcinoma (LUAD) is now the most common histological subtype of primary lung cancer accounting for greater than 40% of cases [7]. Despite achievements in the development of new approaches in the treatment of LUAD, unfortunately, LUAD is still one of the most aggressive and rapidly fatal tumor types with overall survival less than 5 years [8]. Thus, it is important to find stable and reliable biomarkers for LUAD to identify patients with poor prognosis and provide more aggressive treatment [9].

Study identified Cytoplasmic FMR Interacting Protein 2 (CYFIP2) as a p53-inducible gene which was a direct p53 target gene that may be part of a redundant network of genes responsible for p53-dependent apoptosis [10]. During the past decades, accumulating evidence has demonstrated the role of CYFIP2 in different diseases. For obesity and related metabolic disorders, data emphasized the potential of CYFIP2 as a pharmacotherapeutic target for treating obesity and other metabolic disorder [11]. Moreover, CYFIP protein family, including CYFIP1 and CYFIP2, is involved in neural development and maturation as well as in different neural disorders, such as intellectual disabilities, autistic spectrum disorders, and Alzheimer’s disease [12]. Recent studies have shown that CYFIP2 is also closely related to tumor initiation and progression. Fox example, NUAK family kinase 2 (NUAK2) may regulate CYFIP2 expression to promote cervical cancer cell proliferation, migration, invasion and epithelial-to-mesenchymal transition [13]. Circulating CYFIP2 was demonstrated to be significantly upregulated in gastric cancer tissues and cell lines and high expression of circulating CYFIP2 was associated with metastasis and poor prognosis of gastric cancer patients [14]. Studies also indicated that CYFIP2 may take part in the vascular endothelial growth factor receptor signaling pathway and the purine ribonucleoside diphosphate metabolic process [15]. However, the comprehensive function and prognostic landscape of CYFIP2 involved in human cancers are still not fully understood. Thus, understanding its mechanisms may be helpful to enhance diagnosis and treatment and may also promote its potential clinical practice.

**Fig. 1:**
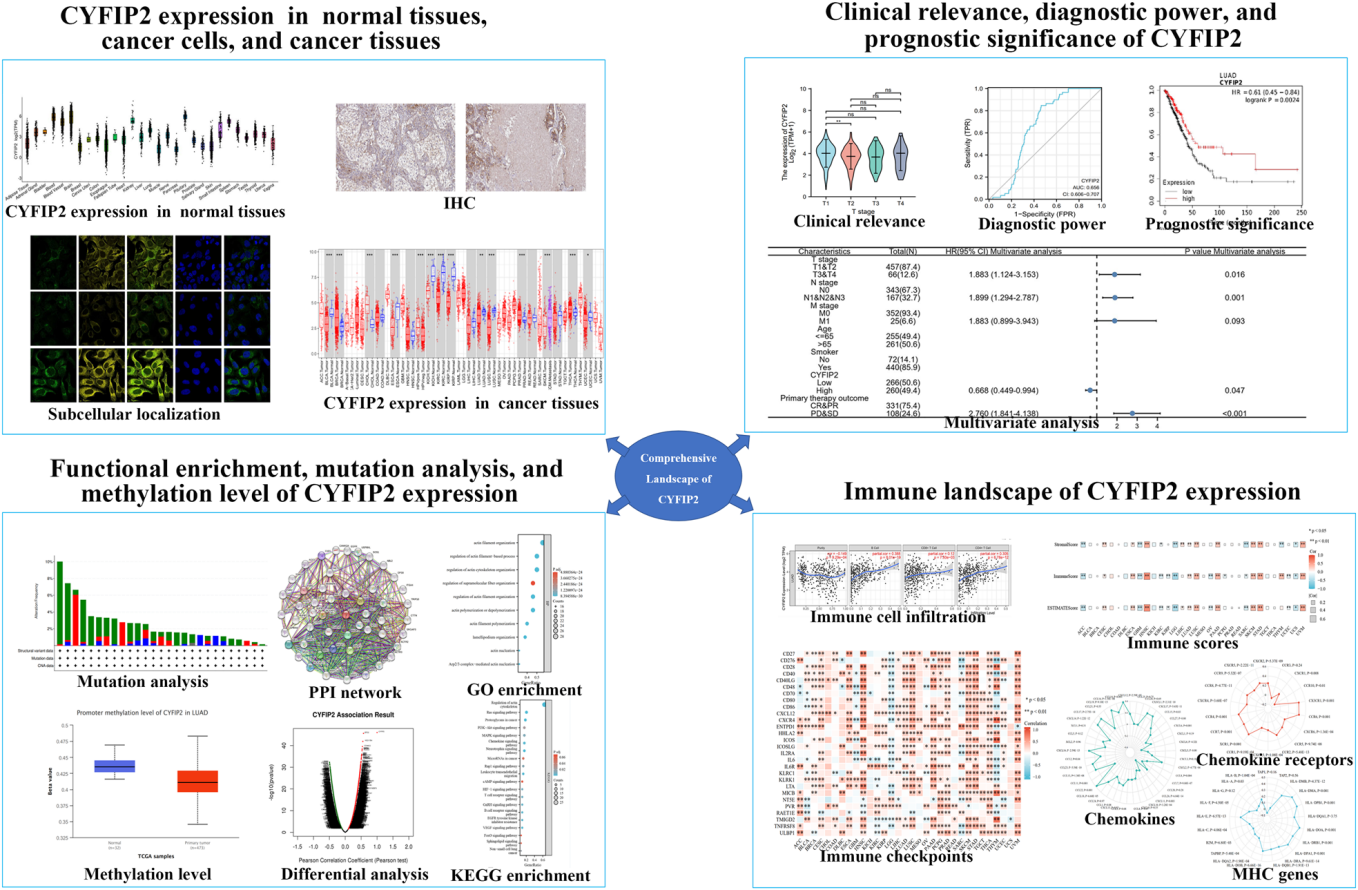
The schematic pipeline for this study

Our study, for the first time, focused on the comprehensive landscape of CYFIP2 function based on a multi-omics analysis. We first revealed the expression patterns including its expression in normal tissues, cancer tissues and intracellular localization. Then, we evaluated the clinical utility of CYFIP2 by studying its associations with different clinicopathological characteristics and potential role in the diagnosis and prognosis of different cancers. Moreover, we investigated the potential molecular mechanisms of CYFIP2 in the pathogenesis or clinical prognosis of different cancers, especially in LUAD, by performing co-expression gene analysis, protein–protein iteration network analysis, functional enrichment analysis, genetic alterations and DNA methylation analysis. In addition, we uncovered the landscape of CYFIP2 expression in tumor immunity and microenvironment through the correlation analysis of CYFIP2 expression with immune cell infiltration, immune regulators, and immune-related genes (chemokines, chemokines receptors, and MHC genes). The schematic pipeline for this study is plotted at Fig. 1.

## Materials and methods

### Expression analysis of CYFIP2

The expression of CYFIP2 in normal tissues was analyzed based on GTEx database [16]. To assess the differences of CYFIP2 in protein expression level, the IHC image was downloaded and analyzed from the HPA database [17]. The intracellular localization of CYFIP2 was also identified by the HPA database. The expression differences of CYFIP2 between cancer and normal tissues in different cancer types were accomplished by the TIMER database [18]. We input CYFIP2 in the “Gene_DE”module of TIMER2 web and observed the expression difference of CYFIP2 between cancer and normal tissues for the different tumors of the TCGA project [19]. We further compared the expression difference of CYFIP2 in LUAD with normal tissues and its paired adjacent tissues.

### Correlations between CYFIP2 and clinical features

Correlations between CYFIP2 and different clinical features such as stage, age, and gender in TCGA-LUAD cohort were analyzed using the Kruskal–Wallis test and visualized by violin plots. Moreover, correlations between CYFIP2 and different clinicopathological characteristics in LUAD were analyzed using the Chi-squared test or Fisher’s exact test and presented in the baseline datasheet.

### Diagnostic analysis and survival analysis of CYFIP2

The diagnostic significance of CYFIP2 in LUAD was estimated using RNA sequencing data from TCGA and illustrated by receiver operating characteristic (ROC) curves using the “pROC” R package. The correlation between CYFIP2 expression and survival in pan-cancer was analyzed by Kaplan–Meier Plotter [20]. The relationship of CYFIP2 expression with overall survival (OS) was analyzed in LUAD, esophageal squamous cell carcinoma (ESCC), bladder carcinoma (BC), Kidney renal clear cell carcinoma (KIRC), Kidney renal papillary cell carcinoma (KIPP), pancreatic ductal adenocarcinoma (PDAC), lung squamous cell carcinoma (LUSC), head and neck squamous cell carcinoma (HNSC), sarcoma, thymoma. Hazard ratios (HRs) with 95% confidence intervals (CIs) and log-rank P-values were calculated. Moreover, multivariable risk analysis with CYFIP2 expression and other variables was performed to explore independent factors.

### Genetic alterations and DNA methylation analysis of CYFIP2

The cBioPortal for Cancer Genomic provides a web resource for exploring, visualizing, and analyzing multidimensional cancer genomics data [21]. Alteration of CYFIP2 status in cancer patients and mutation site were acquired from this database. The UALCAN portal, an easy to use, interactive web-portal to perform to in-depth analyses of TCGA gene expression data, allows us to analyze the promoter methylation level of CYFIP2 in normal and primary tumor tissues [22].

### Co-expression genes analysis

The LinkedOmics database was applied to screen the co-expression genes of CYFIP2 based on the TCGA-LUAD cohort [23]. The top 50 genes significantly related to CYFIP2 were identified and presented in a heat map and volcano plot. Then, the survival maps evaluating the relationships between top 50 genes most positively and negatively associated with CYFIP2 and overall survival (OS)/disease-free survival (DFS) in LUAD were generated.

### PPI analysis and functional enrichment analysis of CYFIP2

The STRING database aims to integrate all known and predicted associations between proteins, including both physical interactions as well as functional associations [24]. In this study, we used STRING to search interacting genes and construct PPI networks. Gene ontology (GO) enrichment and Kyoto Encyclopedia of Genes and Genomes (KEGG) pathway analyses of interacting genes of CYFIP2 were performed by the “ClusterProfiler” package and visualized by the “ggplot2” package [25, 26]. Moreover, significant network module of CYFIP2 PPI network was identified. Survival maps of the relationship between module genes expression and OS/DFS in LUAD were established. In addition, the correlation analyses between the expression of CYFIP2 and these genes in LUAD were performed by Spearman’s correlation analysis and visualized by scatter plot.

### Correlation analysis of CYFIP2 expression and immune cell infiltration

TISIDB is an online web integrated multiple types of data resources in tumor immunology [27]. In this study, we performed TISIDB analysis to determine the expression of CYFIP2 and tumor-infiltrating lymphocytes (TILs) across human cancers. Based on the gene expression profile, the relative abundance of TILs was inferred using gene set variation analysis. The correlations between CYFIP2 and TILs were measured by Spearman’s test. The relationships between CYFIP2 expression and immune infiltration were also determined using the TIMER tool.

### Correlation analysis of CYFIP2 and tumor microenvironment

The tumor microenvironment (TME) is composed of a variety of cells including stromal cells and immune cells, which is significant to efficiently identify appropriate patients for immunotherapies [28]. Correlation analysis of CYFIP2 expression and immune microenvironment scores (stromal score, immune score, and estimate score) were explored with R package “estimate” and presented by scatter plot.

### Correlation analysis of CYFIP2 and immune related genes

The immune regulators and related genes including chemokines, chemokine receptors and MHC genes may play an important role in the tumor initiation and immunity. The correlation analyses of immune regulators and related genes with CYFIP2 were assessed by Spearman’s correlation analysis and visualized by heatmap and radar map. *P*-value < 0.05 was regarded as the significant threshold.

## Results

### Data information

The pan-cancer analysis was carried out in 32 different cancer types. The sample number for each cancer type is listed in Table 1. The TCGA-LUAD cohort was divided into two groups including low- and high- CYFIP2 expression level based on the median expression value. The detailed clinicopathological characteristics are presented at Table 2.

**Table 1:** List of full names and sample numbers for each type of cancer

Dataset	Cancer type	N of samples
TCGA-ACC	adrenocortical carcinoma	79
TCGA-BLCA	bladder urothelial carcinoma	408
TCGA-BRCA	breast invasive carcinoma	1100
TCGA-CESC	cervical squamous cell carcinoma and endocervical adenocarcinoma	306
TCGA-CHOL	cholangiocarcinoma	36
TCGA-COAD	colon adenocarcinoma	458
TCGA-DLBC	diffuse large B-cell lymphoma	48
TCGA-ESCA	esophageal carcinoma	185
TCGA-GBM	glioblastoma multiforme	153
TCGA-HNSC	head and neck squamous cell carcinoma	522
TCGA-KICH	kidney chromophobe	66
TCGA-KIRC	kidney renal clear cell carcinoma	533
TCGA-KIRP	kidney renal papillary cell carcinoma	290
TCGA-LGG	brain lower grade glioma	516
TCGA-LIHC	liver hepatocellular carcinoma	371
TCGA-LUAD	lung adenocarcinoma	539
TCGA-LUSC	lung squamous cell carcinoma	501
TCGA-MESO	mesothelioma	87
TCGA-OV	ovarian serous cystadenocarcinoma	303
TCGA-PAAD	pancreatic adenocarcinoma	179
TCGA-PCPG	pheochromocytoma and paraganglioma	181
TCGA-PRAD	prostate adenocarcinoma	498
TCGA-READ	rectum adenocarcinoma	166
TCGA-SARC	sarcoma	260
TCGA-SKCM	skin cutaneous melanoma	471
TCGA-STAD	stomach adenocarcinoma	415
TCGA-TGCT	testicular germ cell tumors	150
TCGA-THCA	thyroid carcinoma	509
TCGA-THYM	thymoma	120
TCGA-UCEC	uterine corpus endometrial carcinoma	545
TCGA-UCS	uterine carcinosarcoma	57
TCGA-UVM	uveal melanoma	80

**Table 2:** Demographic and clinical characteristics of LUAD patients with low and high expression CYFIP2 in TCGA (n = 539)

Characteristics	Low expression of CYFIP2 (*n* = 269)	High expression of CYFIP2 (*n* = 270)	*P*-value
Pathologic T stage, n (%)			0.007
T1	69 (12.9%)	107 (20%)	
T2	162 (30.2%)	130 (24.3%)	
T3	27 (5%)	22 (4.1%)	
T4	9 (1.7%)	10 (1.9%)	
Pathologic N stage, n (%)			0.404
N0	170 (32.5%)	180 (34.4%)	
N1	51 (9.8%)	46 (8.8%)	
N2&N3	43 (8.2%)	33 (6.3%)	
Pathologic M stage, n (%)			0.795
M0	185 (47.4%)	180 (46.2%)	
M1	12 (3.1%)	13 (3.3%)	
Pathologic stage, n (%)			0.269
Stage I	137 (25.8%)	159 (29.9%)	
Stage II	68 (12.8%)	57 (10.7%)	
Stage III	47 (8.9%)	37 (7%)	
Stage IV	12 (2.3%)	14 (2.6%)	
Gender, n (%)			0.014
Female	130 (24.1%)	159 (29.5%)	
Male	139 (25.8%)	111 (20.6%)	
Age, n (%)			0.003
< = 65	147 (28.3%)	110 (21.2%)	
> 65	116 (22.3%)	147 (28.3%)	
Smoker, n (%)			< 0.001
No	21 (4%)	56 (10.7%)	
Yes	240 (45.7%)	208 (39.6%)	

### The expression patterns of CYFIP2

**Fig. 2:**
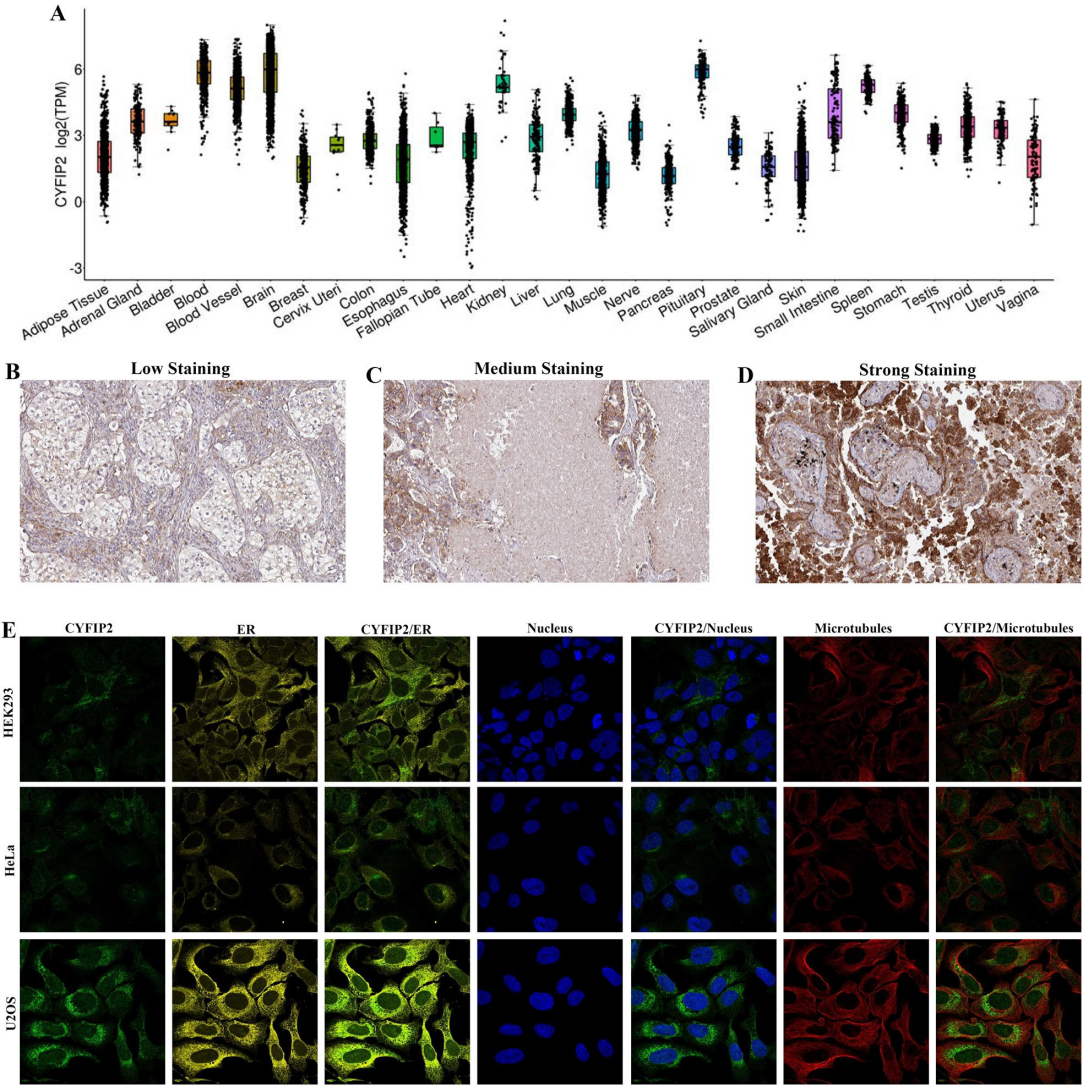
The expression landscape of CYFIP2. **A** The expression of CYFIP2 in normal tissues based on GTEx database. **B**–**D** Representative images of CYFIP2 protein expression of LUAD tissues. **E** Intracellular localization of CYFIP2

To understand the expression and functions of CYFIP2 in different cancers, we should first know its expression in normal tissues. Therefore, the expression level of CYFIP2 in 29 types of normal tissues was first analyzed on the basis of the GTEx database. The results indicated that CYFIP2 expression varied across different types of normal tissues with an expression above medium level of expression in lung tissues (Fig. 2A).

Then, we evaluated the expression of CYFIP2 in tumor tissues using the HPA database. The representative IHC images of CYFIP2 protein expression of LUAD tissues with low-, medium- and strong staining are presented in Fig. 2B–D.

The intracellular localization of CYFIP2 was evaluated in HEK293, HeLa and U2OS cells by immunofluorescent staining of microtubules, endoplasmic reticulum (ER), and nucleus according to the HPA database. Displayed in Fig. 2E, CYFIP2 was found to be localized on the endoplasmic reticulum, plasma membrane and cytosol.

### CYFIP2 is linked with clinical features in different cancer types

**Fig. 3:**
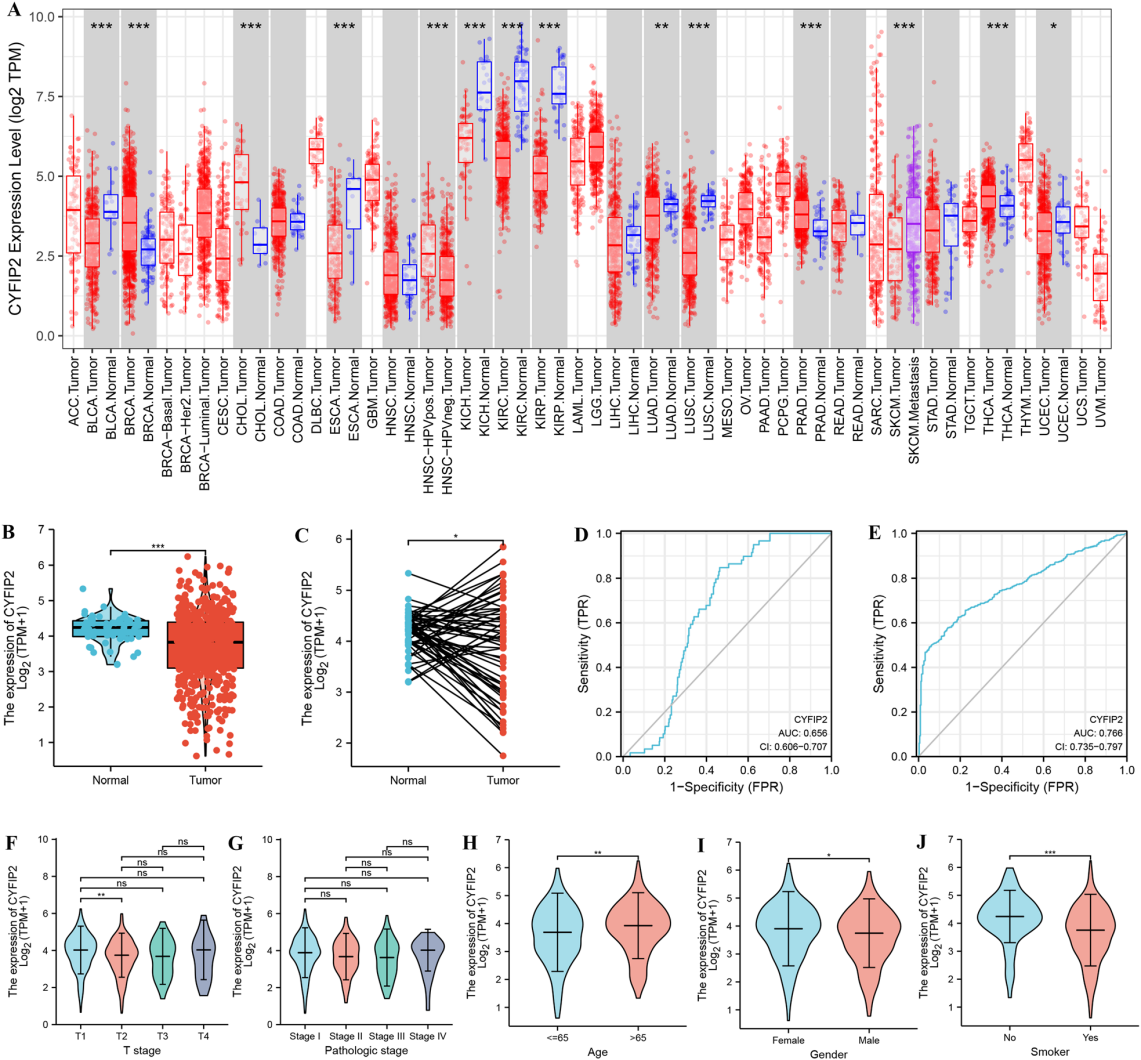
Clinical significance of CYFIP2. **A** CYFIP2 expression levels in tumor tissues and corresponding normal tissues in different cancer types from TCGA data in TIMER 2.0. *P < 0.05, **P < 0.01, ***P < 0.001. **B** CYFIP2 expression levels in LUAD and normal tissues. **C** CYFIP2 expression levels in LUAD and its paired adjacent tissues **D** Receiver operating characteristic analysis of CYFIP2 in LUAD based on TCGA data. **E** Receiver operating characteristic analysis of CYFIP2 in LUAD by combining TCGA and GTEx data. **F**–**J** Association between the CYFIP2 expression and different clinicopathological characteristics in TCGA-LUAD cohort

We then applied the TIMER2 approach to analyze human CYFIP2 expression levels in different cancer types based on TCGA data. As shown in Fig. 3A, the expression levels of CYFIP2 in the tumor tissues of BLCA (*P* < 0.001), ESCA (*P* < 0.001), KICH (*P* < 0.001), KIRC (*P* < 0.001), KIRP (*P* < 0.001), LUAD (*P* < 0.01), LUSC (*P* < 0.001), and UCEC (*P* < 0.05) were significantly lower than the corresponding control tissues. In contrast, the expression levels of CYFIP2 in the tumor tissues of BRCA (*P* < 0.001), CHOL (*P* < 0.001), PRAD (*P* < 0.001) and THCA (*P* < 0.001) were significantly higher than the corresponding control tissues.

We further explored the clinical significance of CYFIP2 expression in LUAD. The results showed that CYFIP2 was markedly downregulated in LUAD tumor tissue compared with normal tissue (Fig. 3B) and paired adjacent normal tissues (Fig. 3C). The diagnostic potential of CYFIP2 in LUAD was then evaluated. ROC analysis indicated that the AUC of CYFIP2 expression in diagnosing LUAD was 0.656 (95% CI 0.606–0.707) based on TCGA data (Fig. 3D). Interestingly, when combining TCGA and GTEx data (Fig. 3E), the diagnostic power of CYFIP2 in LUAD became more significant with an AUC of 0.766 (95% CI 0.735–0.797). Furthermore, we found that CYFIP2 expression was correlated with some clinicopathological characteristics such as T stages, age, gender, and smoking status while no significant association was detected with pathologic stages (Fig. 3F–J).

### CYFIP2 is associated with survival outcomes in different cancer types

**Fig. 4:**
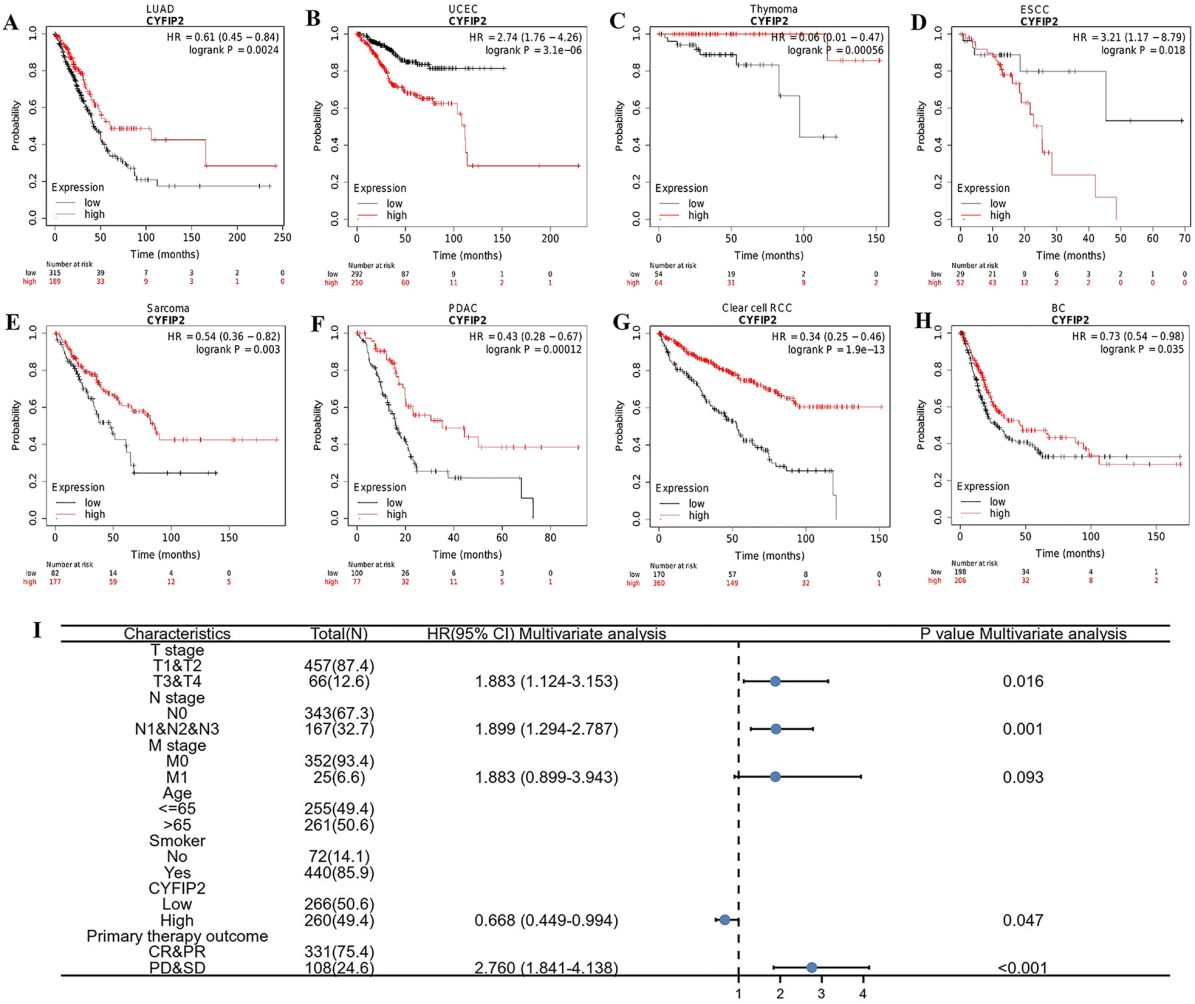
Prognostic value of CYFIP2. (A-H) Kaplan–Meier survival curves of OS comparing the high and low expression of CYFIP2 in different types of cancer including **A** LUAD, **B** UCEC, **C** THYM, **D** ESCC, **E** SARC, **F** PDAC, **G** Clear cell RCC, and **H** BC. **I** The forest plot of multivariate regression analysis result of CYFIP2 in LUAD

We used Kaplan–Meier Plotter, which is mainly based on Affymetrix microarray information from TCGA, to assess CYFIP2-related OS in different cancers (Fig. 4A–H). High expression of CYFIP2 was identified as a favorable prognostic factor in LUAD (HR = 0.61, 95% CI 0.45–0.84, log-rank *P* < 0.01), thymoma (HR = 0.06, 95% CI 0.01–0.47, log-rank P < 0.01), sarcoma (HR = 0.54, 95% CI 0.36–0.82, log-rank *P* < 0.01), PDAC (HR = 0.43, 95% CI 0.28–0.67, log-rank *P* < 0.01), clear cell RCC (HR = 0.34, 95% CI 0.25–0.46, log-rank *P* < 0.01), BC (HR = 0.73, 95% CI 0.54–0.98, log-rank *P* = 0.035). For UCEC and ESCC, CYFIP2 was found to have an unfavorable effect on OS (HR = 2.74, 95% CI 1.76–4.26, log-rank *P* < 0.01; HR = 3.21, 95% CI 1.17–8.79, log-rank *P* = 0.018).

We further explored the potential relationship between CYFIP2 expression and several clinical features of patients with LUAD (Fig. 4I). The results of multivariate regression analyses showed that high expression of CYFIP2 played a benefited role in patients with LUAD (HR = 0.668, 95% CI 0.449–0.994, *P* = 0.047). Some characteristics also played a detrimental role in patients with LUAD, such as T3&T4 stage (HR = 1.883, 95% CI 1.124–3.153, *P* = 0.016), lymph node-positive disease (HR = 1.899, 95% CI 1.294–2.787, *P* = 0.001), progressive disease (PD) & stable disease (SD) (HR = 2.760, 95% CI 1.841–4.138, P < 0.001).

### CYFIP2 is mutated with missense in different cancer types

**Fig. 5:**
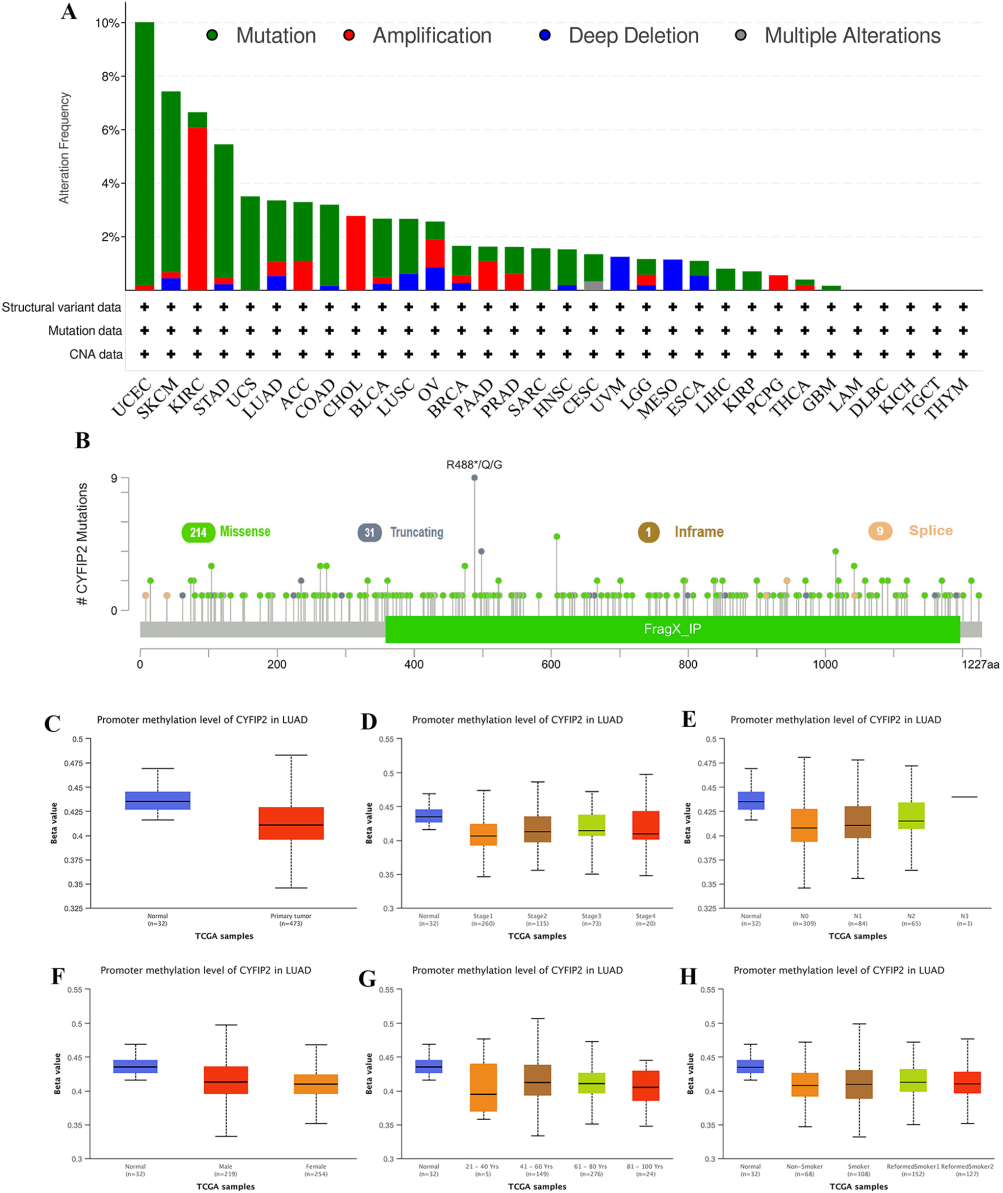
Mutation and methylation analysis results of CYFIP2. **A** Mutation feature of CYFIP2 in different tumors of TCGA using the cBioPortal tool. **B** The mutation sites of CYFIP2. **C** Promoter methylation levels of CYFIP2 in LUAD between normal and tumor tissues. **D** Promoter methylation levels of CYFIP2 in LUAD among different clinical stages. **E** Promoter methylation levels of CYFIP2 in LUAD among different N stages. **F** Promoter methylation levels of CYFIP2 in LUAD among between male and female. **G** Promoter methylation levels of CYFIP2 in LUAD among different ages. **H** Promoter methylation levels of CYFIP2 in LUAD among different smoking status

We performed comparative analysis of CYFIP2 using cBioPortal database to figure out genomic mutation of CYFIP2 in different cancers (Fig. 5A). The genetic alteration profiling of CYFIP2 showed that its mutation is one of the most important single factors for alteration in UCEC, SKCM, STAD, UCS, LUAD, ACC, COAD, BLCA, and LUSC. In addition, CYFIP2 amplification frequencies are the highest in KIRC, CHOL, PAAD, and PCPG. The types, sites and case numbers of the CYFIP2 genetic alteration are further presented in Fig. 5B. Missense mutation of CYFIP2 is the main type of genetic alteration.

### CYFIP2 mRNA expression correlates with hypomethylation

DNA methylation has been recognized as one of the most important ways in the regulation of gene transcription, and thus we supposed that dysregulation of CYFIP2 expression may be regulated in this way. Accordingly, as shown in Fig. 5C, the results revealed that gene methylation negatively correlated with CYFIP2 gene expression (*P* < 0.05) in TCGA-LUAD cohort. In addition, the promoter methylation of CYFIP2 in tumor tissues of TCGA-LUAD was significantly lower than that of normal tissues adjacent to the cancer regardless of different clinical stages, N stages, gender, age and smoking status (Fig. 5D–H).

### CYFIP2 has a synergistic effect with its co-expressed genes

**Fig. 6:**
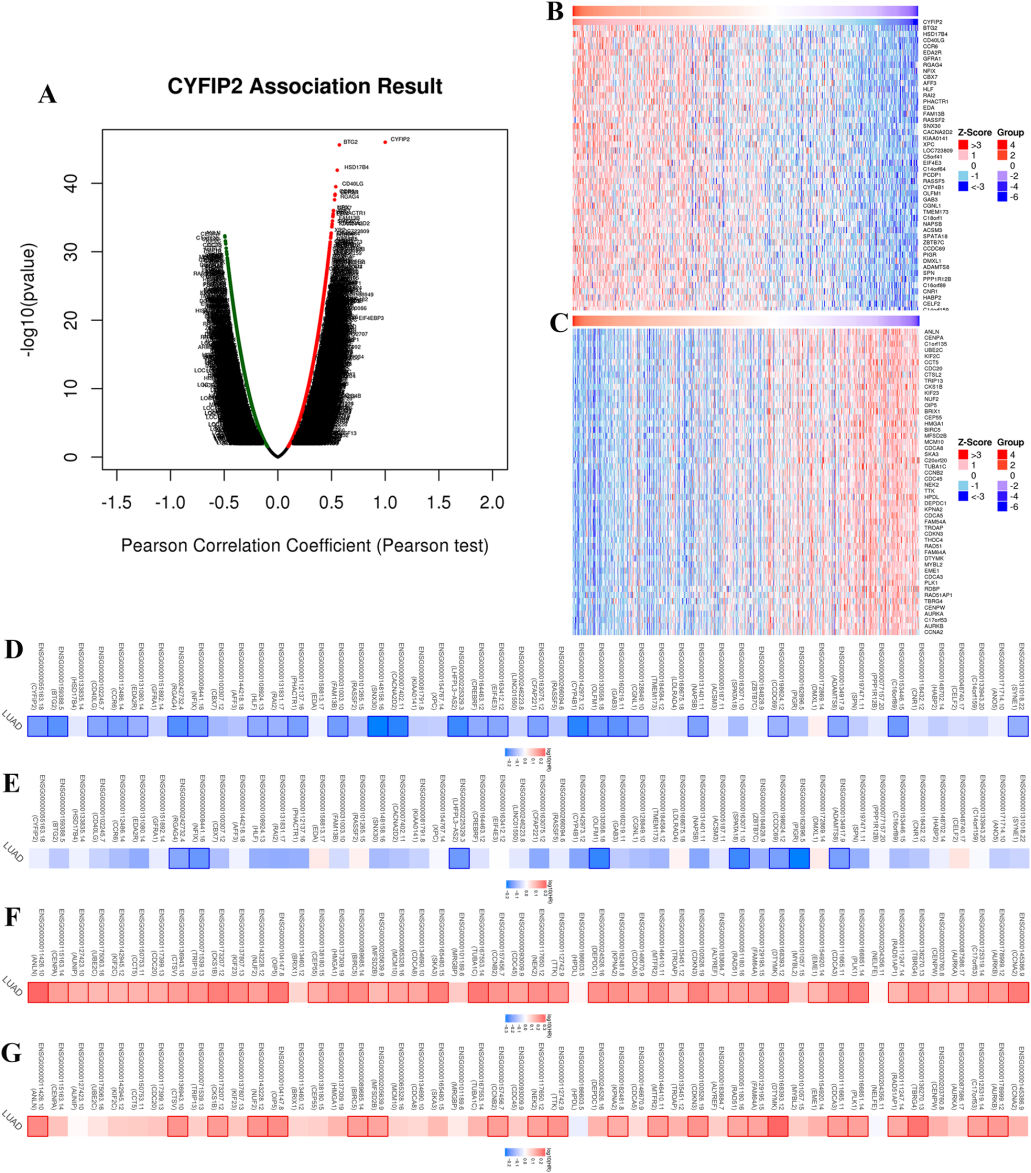
The co-expression genes with CYFIP2 in TCGA-LUAD cohort. **A** The whole significantly associated genes with CYFIP2 distinguished by Pearson test in the TCGA-LUAD cohort. **B** The top 50 genes positively related to CYFIP2 in the TCGA-LUAD cohort by heat map. **C** The top 50 genes negatively related to CYFIP2 in the TCGA-LUAD cohort by heat map. **D** Survival maps of the relationship between top 50 genes most positively associated with CYFIP2 and OS in LUAD. **E** Survival maps of the relationship between top 50 genes most positively associated with CYFIP2 and DFS in LUAD. **F** Survival maps of the relationship between top 50 genes most negatively associated with CYFIP2 and OS in LUAD. **G** Survival maps of the relationship between top 50 genes most negatively associated with CYFIP2 and DFS in LUAD

For gaining the in-depth knowledge of CYFIP2 biological function in LUAD, we used the LinkedOmics database to study the correlated significant genes based on TCGA-LUAD cohort. The whole genes significantly related CYFIP2 are identified and presented in the volcano plot (Fig. 6A). As shown in the heat map (Fig. 6B, C), the top 50 marked genes were positively and negatively associated with CYFIP2. We further explored the prognostic role of the marked genes positively and negatively related to CYFIP2. As plotted by the survival maps (Fig. 6D–G), most of the top 50 positively correlated genes had favorable protective HRs for both OS and DFS in LUAD, while nearly all the negatively correlated genes had an unfavorable protective HR for both OS and DFS, suggesting these co-expressed genes highly owned the probability of becoming potential biomarkers in LUAD. These results indicated that CYFIP2 may have a synergistic effect with its co-expressed genes in the prognosis of LUAD.

### CYFIP2 is implicated in the regulation of signaling pathways

**Fig. 7:**
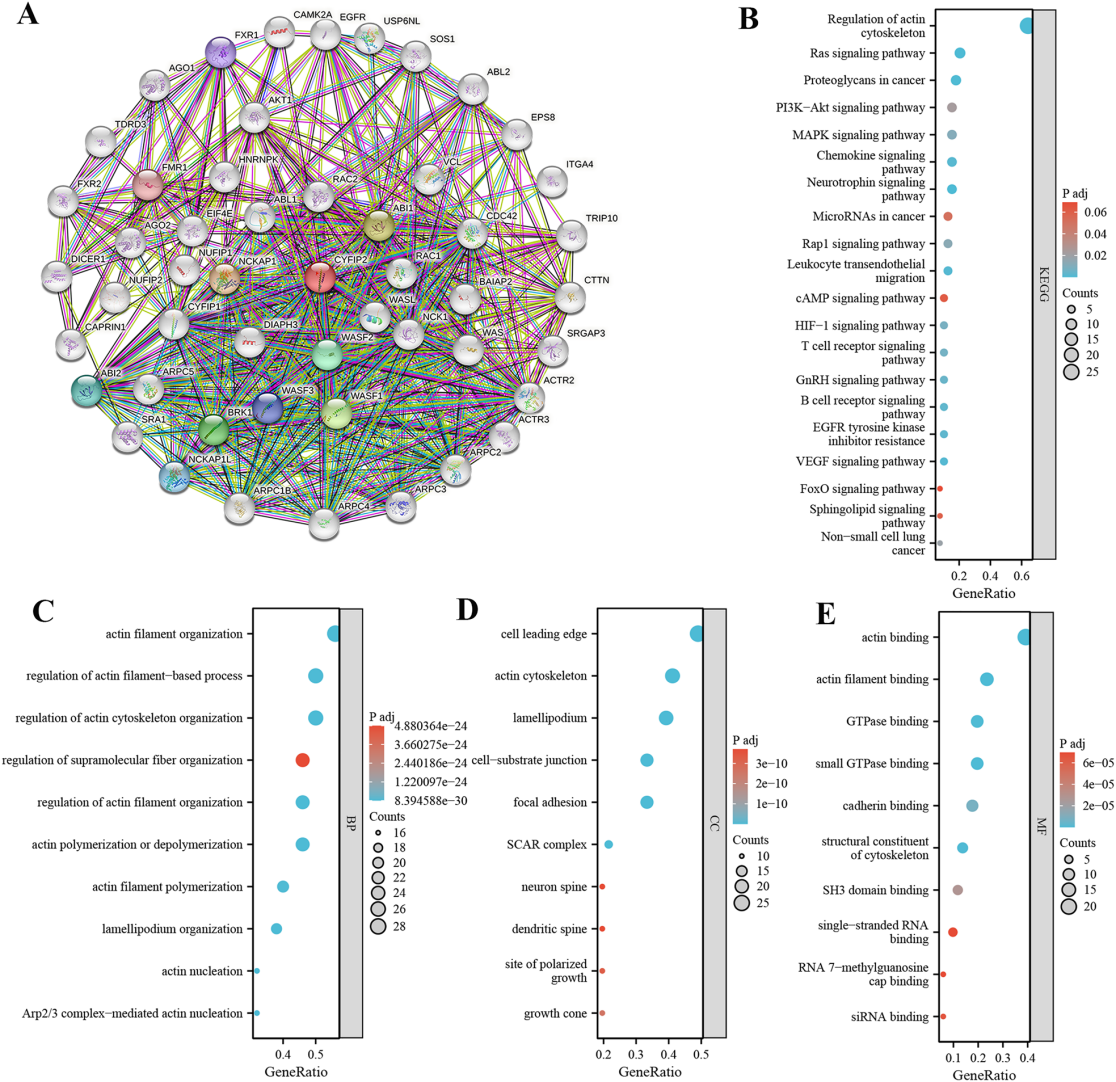
CYFIP2 protein–protein network and functional enrichment analysis. **A** PPI network for CYFIP2-interaction genes. **B** KEGG pathway analysis. **C** Biological process of GO analysis. **D** Cellular component of GO analysis. **E** Molecular function of GO analysis

CYFIP2 may also interact closely with some genes in the initiation and progression of different cancers. STRING database was used to construct an integrated PPI network of CYFIP2-interacted genes and performed functional annotations through GO and KEGG analyses. Figure 7A shows a PPI network of CYFIP2 and its top 50 interacted genes. As shown in Fig. 7B, CYFIP2-interacting genes were highly involved in a series of LUAD and immunity related pathways such as Ras signaling pathway, PI3K/AKT signaling pathway, VEGF signaling pathway, chemokine signaling pathway, EGFR tyrosine kinase inhibitor resistance, B cell receptor signaling pathway, T cell receptor signaling pathway, HIF-1 signaling pathway, and non-small cell lung cancer.

Consistent with the KEGG enrichment results, the CYFIP2-interacting genes were highly enriched in a series of important GO functional annotations involved in tumor initiation and progression. In the BP category (Fig. 7C), these genes were significantly enriched in actin filament organization, regulation of actin filament-based process, and actin filament polymerization. The cell leading edge, actin cytoskeleton, lamellipodium, cell-substrate junction, focal adhesion, SCAR complex, neuron spine, dendritic spine, site of polarized growth, and growth cone were the top ten most significantly enriched items in the CC category (Fig. 7D). In terms of MF, the GO terms were highly involved in processes including actin binding, small GTPase binding, and GTPase binding (Fig. 7E).

**Fig. 8:**
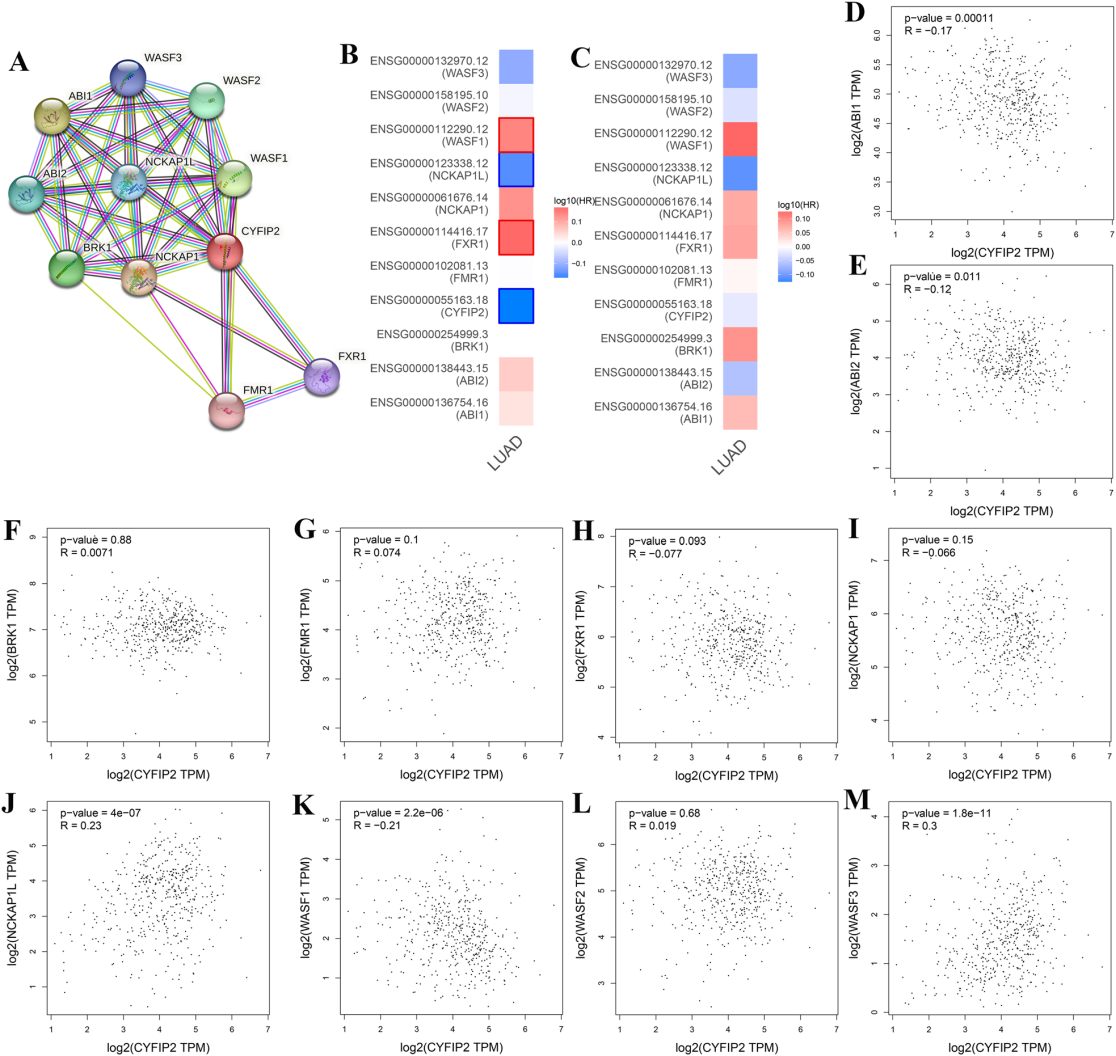
Sub-network and significant CYFIP2-interacting proteins. **A** A significant network module of CYFIP2 and its interacting genes. **B** Survival maps of the relationship between CYFIP2-interacting proteins expression and OS in LUAD. **C** Survival maps of the relationship between CYFIP2-interacting proteins expression and DFS in LUAD. **D**–**M** The correlation analyses between the expression of CYFIP2 and co-expressed genes in LUAD

Furthermore, a significant module with top ten interacting genes was identified (Fig. 8A). We further evaluated the prognostic value of these genes in LUAD. The survival maps indicated that the top ten CYFIP2-interacting genes may also have a predictive role in the OS and DFS of LUAD (Fig. 8B, C). Scatter plots by Spearman’s correlation analysis further presented the correlations between CYFIP2 expression and the top ten CYFIP2-interacting genes based on the TCGA-LUADA cohort (Fig. 8D–M). In LUAD, the expression of CYFIP2 was negatively correlated with the expression of ABI1 (r = − 0.17, *P* < 0.001), ABI2 (*r* = − 0.12, *P* = 0.011), and WASF1 (r = − 0.21, *P* < 0.001), while the expression of CYFIP2 was positively correlated with the expression of NCKAP1L (*r* = 0.23, *P* < 0.001) and WASF3 (*r* = 0.3, *P* < 0.001).

### CYFIP2 is strongly associated with the infiltration of immune cells

**Fig. 9:**
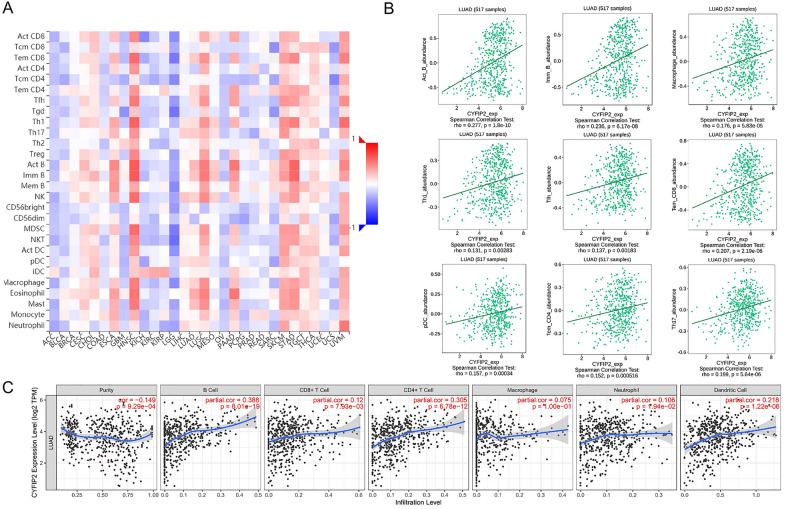
Correlation between CYFIP2 expression of immune infiltration levels. **A** Relations between the expression of CYFIP2 and 28 types of TILs across human cancers. **B** Correlation of CYFIP2 expression with abundance of Act_B cells, Imm_B cells, macrophage cells, Th1 cells, Tfh cells, CD8 + cells, pDC cells, CD4 + cells and Th17 cells. **C** Correlation of CYFIP2 expression with immune infiltration level in LUAD. CYFIP2 expression has significant negative relation with tumor purity and significant positive correlation with infiltrating levels of B cell, CD8 + T cell, CD4 + T cell, macrophage, neutrophil, and dendritic cell. LUAD, lung adenocarcinoma. *P* < 0.05 is considered as significant

Since CYFIP2 participated in regulating immune-related pathways, we further analyzed the correlations between CYFIP2 expression and 28 types of TILs by the TISIDB database. Figure 9A displays the relationships between expression of CYFIP2 and 28 types of TILs across different cancers. Figure 9B presents that the expression of CYFIP2 was correlated with abundance of act_B cells (*r* = 0.277, *P* = 1.8e–10), imm_B cells (*r* = 0.236, *P* = 6.17e–8), macrophage cells (*r* = 0.176, *P* = 5.83e–5), Th1 cells (*r* = 0.131, *P* = 2.83e–3), Tfh cells (*r* = 0.137, *P* = 1.83e–8), CD8 + cells (r = 0.207, *P* = 2.19e-6), pDC cells (*r* = 0.157, *P* = 3.4e–4), CD4 + cells (*r* = 0.152, *P* = 5.16e–4) and Th17 cells (*r* = 0.199, *P* = 5.64e–6).

Using the TIMER database, we further evaluated the relationships between CYFIP2 expression and six main types of tumor infiltrating immune cells of LUAD. As shown in Fig. 9C, CYFIP2 expression had significantly negative associations with tumor purity (*r* = − 0.149, *P* = 9.29e-04) and significantly positive correlations with infiltrating levels of B cell (*r* = 0.388, *P* = 8.01e–19), CD8 + T cell (*r* = 0.12, P = 7.93e–03), CD4 + T cell (*r* = 0.305, *P* = 6.78e–12), macrophage (*r* = 0.075, *P* = 1.00e–1), neutrophil (*r* = 0.106, *P* = 1.94e–02), and dendritic cell (*r* = 0.218, *P* = 1.22e–06).

**Fig. 10:**
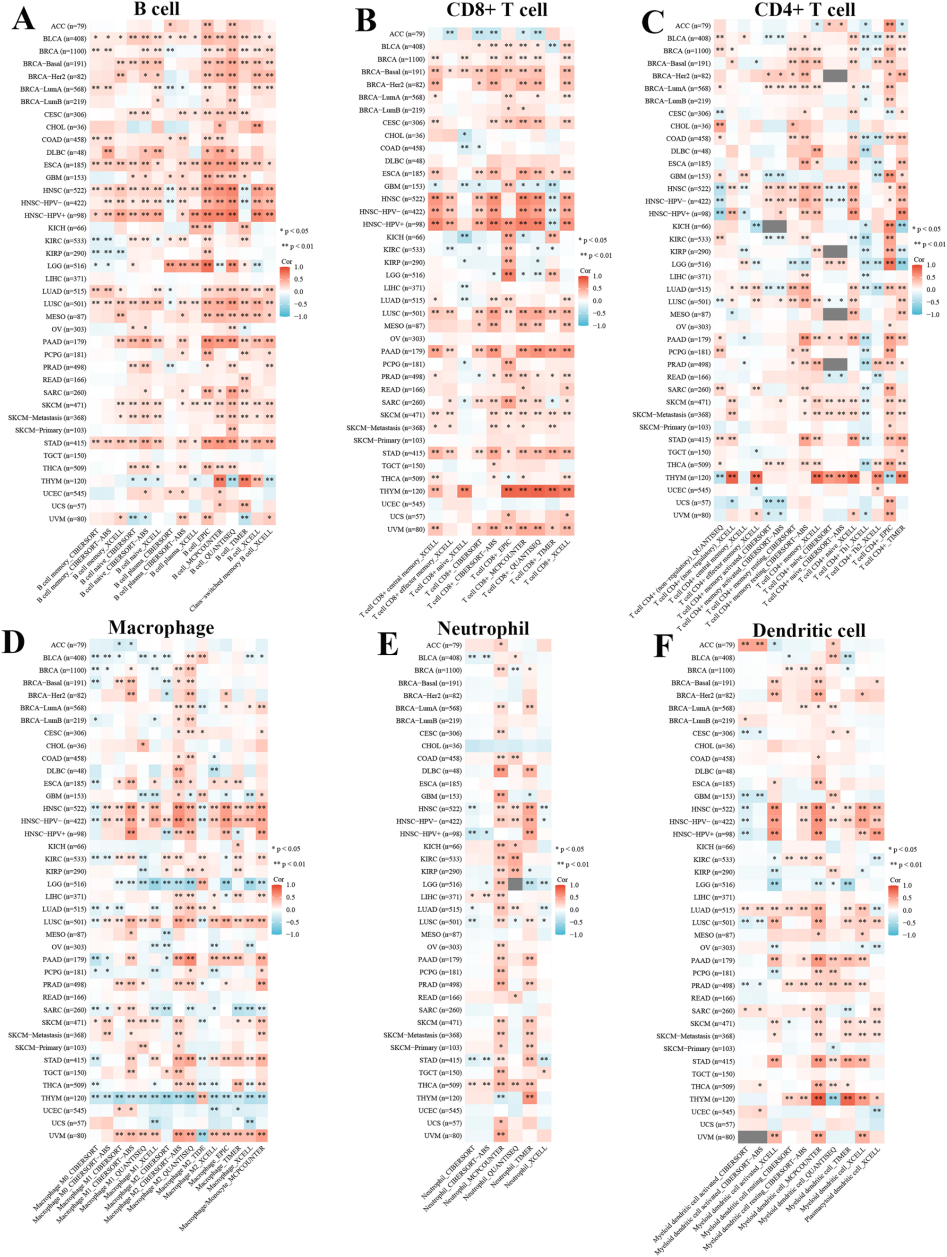
Correlation of CYFIP2 expression with the infiltration of immune cells in different cancers. **A** The infiltration of B cell; **B** The infiltration of CD8 + T cells; **C** The infiltration of CD4 + T cells; **D** The infiltration of macrophages; **E** The infiltration of neutrophils; **F** The infiltration of dendritic cells

Moreover, different algorithms, including CIBERSORT, CIBERSORT-ABS, XCELL, TIMER, EPIC, QUANTISEQ, and MCPCOUNTER, were employed to further investigate the correlations between the above six tumor infiltrating immune cells and CYFIP2 expression in different cancers. As shown in Fig. 10, the results revealed that CYFIP2 expression was positively correlated with B cells, CD8 + T cells, CD4 + T cells, macrophages, neutrophils, and dendritic cells in most cancer types based on all or most algorithms, especially in LUAD. These results highly suggested that CYFIP2 played a vital part in regulating the infiltration levels of different immune cells.

### CYFIP2 is highly involved in the tumor immune microenvironment

**Fig. 11:**
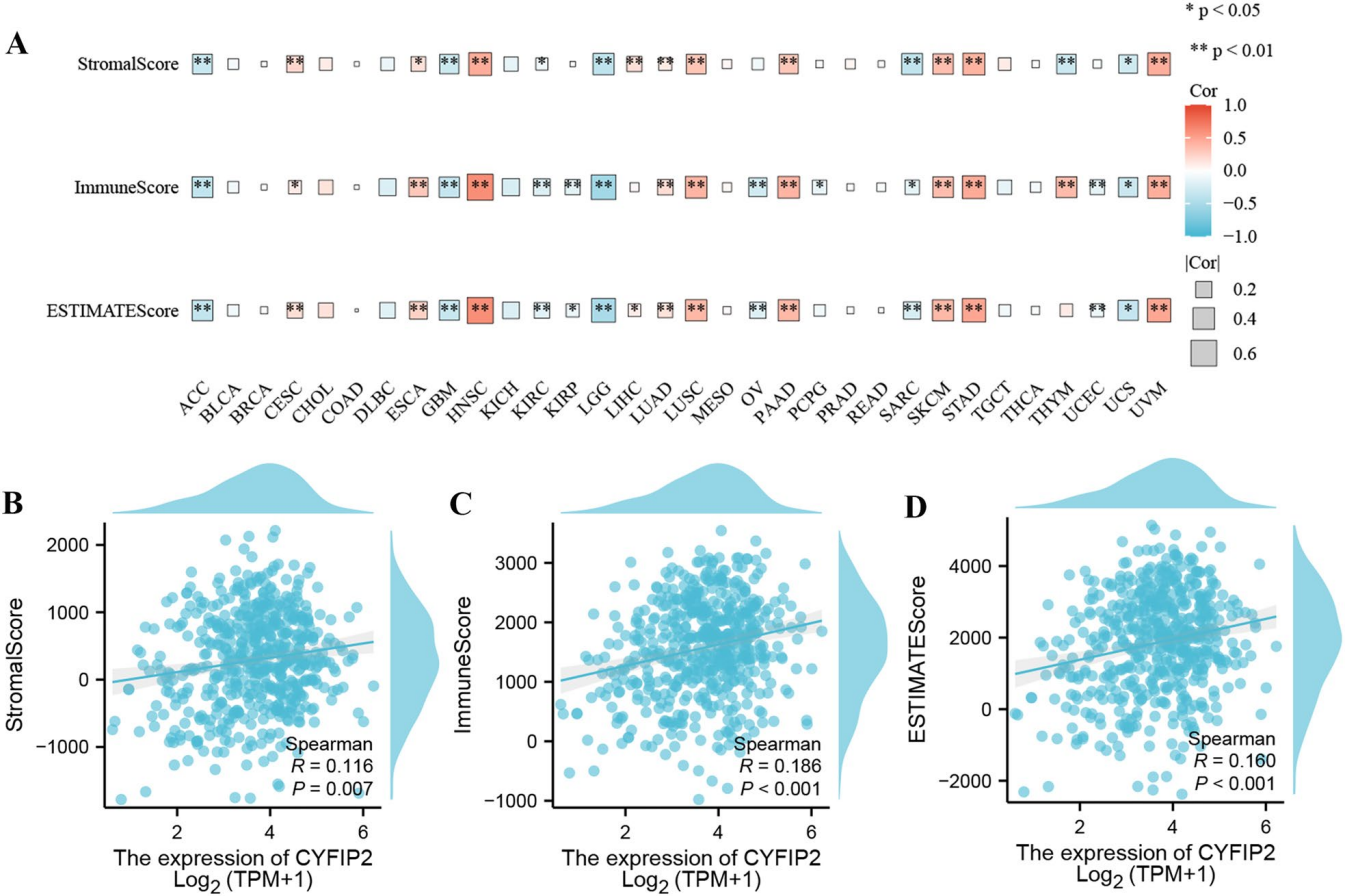
Correlation between CYFIP2 and immune microenvironment scores. **A** The expression correlation between CYFIP2 and immune microenvironment scores in different cancers by heatmap. **B** The expression correlation between CYFIP2 and stromal score in LUAD by scatter chart. **C** The expression correlation between CYFIP2 and immune score in LUAD by scatter chart. **D** The expression correlation between CYFIP2 and estimate score in LUAD by scatter chart

For tumor microenvironment analysis, the R package “estimate” was applied to calculate the immune score, stromal score and estimate score based on the CYFIP2 expression level. The expression correlations between CYFIP2 and immune microenvironment scores in different cancers were plotted by heatmap in Fig. 11A. The results revealed that the stromal score, immune score and estimate score of ACC, GBM, KIRC, LGG, SARC, and UCS were negatively associated with CYFIP2 expression levels. In addition, the stromal score, immune score and estimate score of CESC, ESCA, HNSC, LUAD, LUSC, PAAD, SKCM, STAD, and UVM were positively associated with CYFIP2 expression levels. As shown in Fig. 11B-D, all tumor immune microenvironment scores of LUAD were significantly related to the CYFIP2 expression level.

### CYFIP2 is significantly related to immune regulators and related genes

**Fig. 12:**
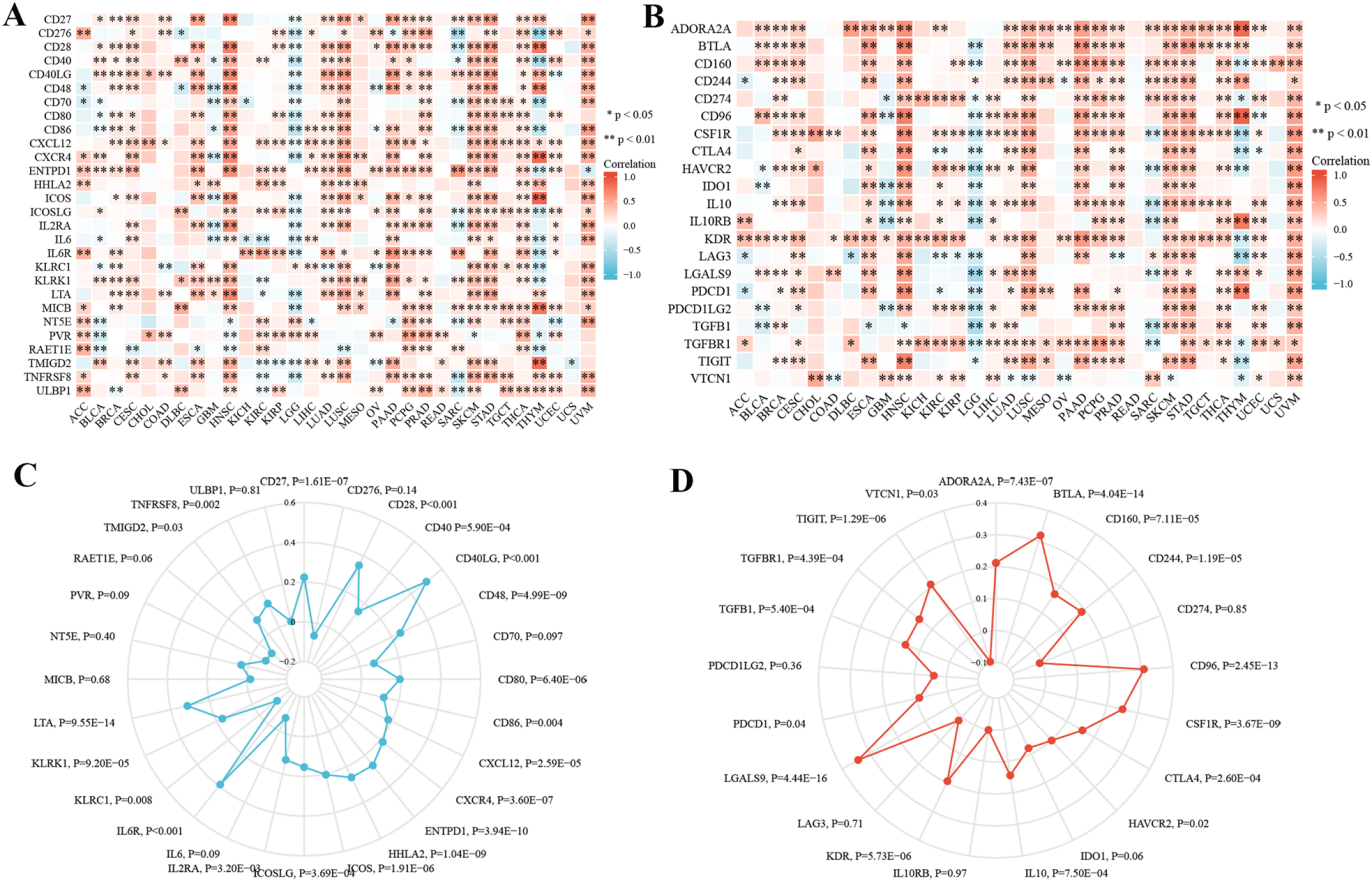
Correlation between CYFIP2 and immune regulator expression. **A** The expression correlation between CYFIP2 and immune stimulators in different cancers by heatmap. **B** The expression correlation between CYFIP2 and immune inhibitors in different cancers by heatmap. **C** The expression correlation between CYFIP2 and immune stimulators in LUAD by radar chart. **D** The expression correlation between CYFIP2 and immune inhibitors in LUAD by radar chart

**Fig. 13:**
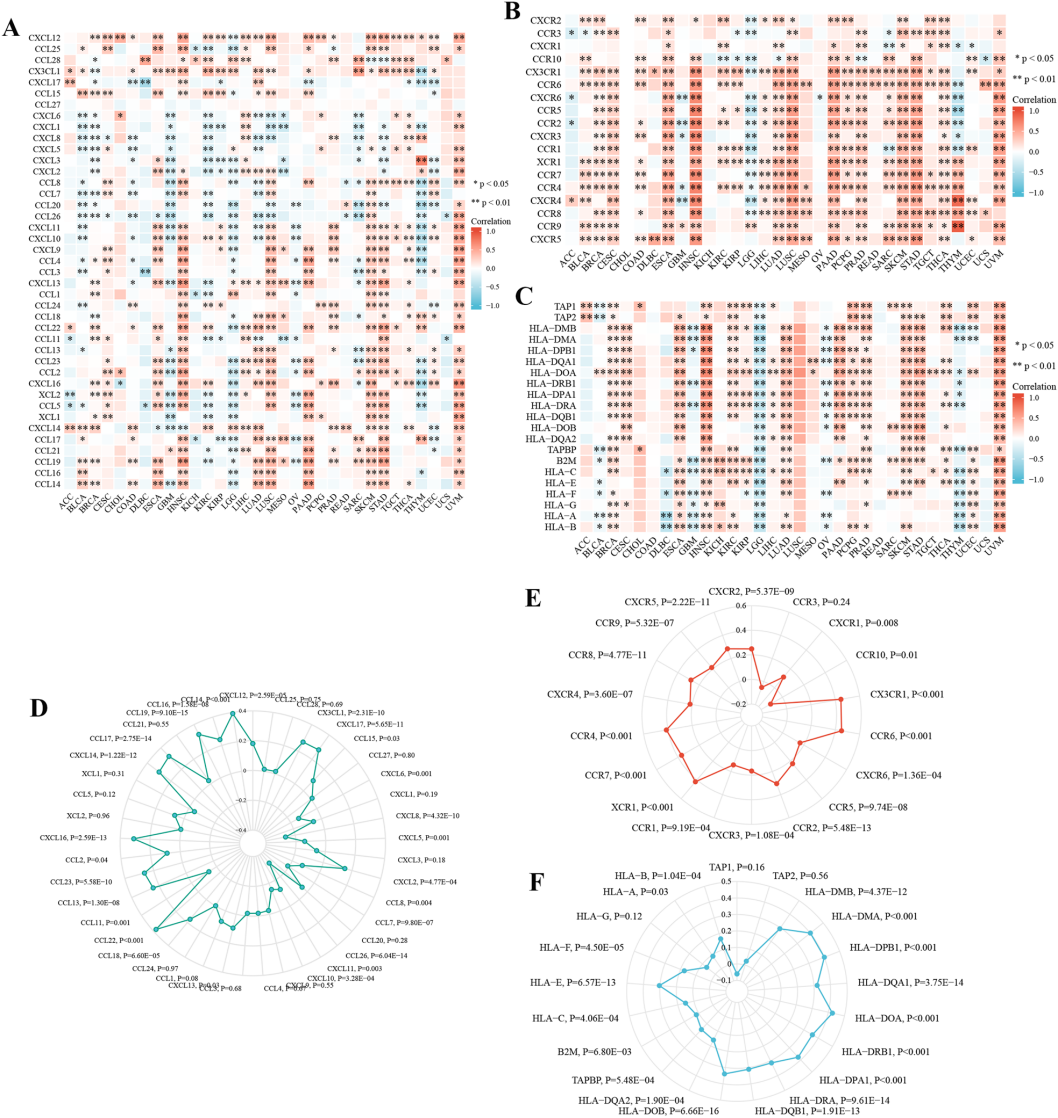
Correlation between CYFIP2 and immune related genes. **A** The expression correlation between CYFIP2 and chemokines in different cancers by heatmap. **B** The expression correlation between CYFIP2 and chemokine receptors in different cancers by heatmap. **C** The expression correlation between CYFIP2 and MHC genes in different cancers by heatmap. **D** The expression correlation between CYFIP2 and chemokines in LUAD by radar chart. **E** The expression correlation between CYFIP2 and chemokine receptors in LUAD by radar chart. **F** The expression correlation between CYFIP2 and MHC genes in LUAD by radar chart

As shown in Fig. 12A, B, CYFIP2 was positively associated with most immune stimulators and immune inhibitors in different cancers. Figure 12C, D illustrates the detailed expression correlations between CYFIP2 and immune stimulators/inhibitors. In addition, CYFIP2 expression was also highly related to almost most chemokines, chemokine receptors, and MHC genes in different cancers (Fig. 13A-C). The detailed expression correlations between CYFIP2 and chemokines/chemokine receptors/MHC genes were presented at Fig. 13D–F. These results suggested that CYFIP2 might regulate immune cell infiltration via these genes.

## Discussion

CYFIP2, a p53-inducible gene, has been demonstrated by previous studies to be involved in some tumors. Jiao et al. reported CYFIP2 was a novel p53-mediated pro-apoptotic protein whose expression was decreased in gastric cancer and they showed CYFIP2 knockdown promoted proliferation and colony formation, and inhibited apoptosis in gastric cancer cells [29]. Chang’s study revealed that mutational dynamics and genetic variation, as well as aberrant DNA repair, tumor cell cycle control and apoptotic pathways were associated with CYFIP2 in endometrial cancer in the Taiwanese population [30]. Previous studies also showed that CYFIP2 was downregulated in ccRCC patients with an unfavorable survival outcome and might be a potential promising prognostic biomarker associated with immune infiltration, metabolism, and epithelial–mesenchymal transition process in ccRCC [31]. However, the associations between CYFIP2 and different cancers remain inclusive. Besides, the specific mechanisms of CYFIP2 involved in cancer initiation and progression remain to be unclear. The present study first comprehensively uncovered the landscape of CYFIP2 regarding its expression pattern, clinical relevance, genetic alterations, DNA methylation and immune cell infiltration.

We first explored the expression patterns in different levels including normal tissues, cell lines and tumor tissues. Our study demonstrated that CYFIP2 was aberrantly expressed in different cancers. The difference in CYFIP2 expression levels in different tumor types may reflect distinct underlying functions and mechanisms. In addition, CYFIP2 was identified to have the potential for diagnosis of LUAD. We further found that overexpression of CYFIP2 generally predicted good prognosis for patients with tumors, such as LUAD, thymoma, sarcoma, PDAC, clear cell RCC, and BC. In contrast, its highly expression was correlated with poor prognosis in UCEC and ESCC. In addition, a high level of CYFIP2 expression was shown to be related to a favorable prognosis in LUAD with T1&T2 stage, N0 stage, CR & PR diseases. These results suggest that CYFIP2 may serve as a potential biomarker for diagnosis and predicting the prognosis of tumor patients.

Genetic alterations in the key genes have been demonstrated to be highly involved in the initiation and progression of cancer through regulating important signaling pathways that are indispensable for the fundamental intracellular functions including cell growth, survival, invasion, and metastasis [32]. The genetic alteration profiling of CYFIP2 revealed that its mutation is the most important single factors. The impact of mutant subtypes on prognosis and predictive value in LUAD is becoming increasingly understood [33]. Knowledge of genetic alterations will provide more useful information for the diagnosis, treatment and prognosis of cancer.

DNA methylation is a major form of epigenetic modification and is involved in numerous fundamental biological activities through post-transcriptional regulation of gene expression [34]. Aberrant patterns of DNA methylation can lead to the pathogenesis of many diseases including various cancers [35]. Hypermethylation within the promoter region may contribute to silencing or inactivation of tumor suppressor genes in cancer cells [36]. In addition, recent evidence has indicated that DNA methylation has also important roles in the immune system and influence tumor immunotherapy response as DNA methylation is essential for the proper function of B-cell differentiation and mature cells [37]. The present study showed that DNA methylation of CYFIP2 was downregulated in LUAD, which is consistent with the fact that CYFIP2 had a protective role for LUAD patients.

In complex diseases, a specific gene generally plays a coordinating role through its co-expressed and interacting genes. Our results also indicated that CYFIP2 may have a synergistic effect with its co-expressed genes in the prognosis of LUAD. Moreover, our KEGG analysis revealed that CYFIP2-interacting genes were strongly involved in a series of tumor-related and immune-related signaling pathways. For example, CYFIP2-interacting genes were remarkedly linked with non-small cell lung cancer, which was consistent with our present study that CYFIP2 took part in the initiation and progression of LUAD. CYFIP2-interacting genes were also strongly related to Ras signaling, which is a well-studied pathway that is involved in cell proliferation and growth, cell survival and apoptosis, metabolism, and motility and plays an important role in tumor growth and progression [38]. VEGF signaling pathway and EGFR tyrosine kinase inhibitor resistance are two important pathways for the targeted therapy of LUAD [39, 40]. The PI3K/Akt/mTOR pathway has been one of the most altered molecular pathways implicated in both tumorigenesis and the progression of LUAD [41]. Recent evidence also revealed that the PI3K/AKT/mTOR pathway can play a vital role in regulating PD-L1 expression, the abnormal activation of which can contribute to the increased PD-L1 protein translation [42]. In addition, CYFIP2-interacting genes were significantly associated with some immune-related pathways such as chemokine signaling pathway, B cell receptor signaling pathway, and T cell receptor signaling pathway, which play a vital role of tumor immunity and immunotherapy response [43–45]. These results confirmed that CYFIP2 played an important role in the tumor development and immune microenvironment.

The immune cells within the TME play important roles in tumorigenesis [46]. It has been known that these tumors associated immune cells may possess tumor-antagonizing or tumor-promoting functions [47]. Tumor-infiltrating immune cells as well as other immune components within the TME can shape an immunosuppressive TME that enables cancer cells to evade surveillance of the immune system [48]. Immune cells are important constituents of the tumor stroma. Studies suggested that the innate immune cells as well as adaptive immune cells contribute to tumor progression when present in the TME [47]. Our study showed the correlation analysis between CYFIP2 expression and immune cell infiltration in pan-cancer. We found that several tumor infiltrating immune cells (Act_B cells, Imm_B cells, macrophage cells, Th1 cells, Tfh cells, CD8 + cells, pDC cells, CD4 + cells and Th17 cells) were correlated with the expression of CYFIP2 in cancers using TISIDB. We further found that positive correlation was indicated between CYFIP2 expression and B cell, CD8 + T cell, CD4 + T cell, macrophage, neutrophil, and dendritic cell in LUAD using TIMER. This finding suggests that there is a potential correlation between CYFIP2 and immune infiltration in LUAD.

Besides that, the CYFIP2 expression presented a significantly correlation with most immune regulators, chemokines, chemokine receptors, and MHC genes. Emerging evidence has supported that immune regulators play a crucial part in establishing an immunosuppressive TME, causing NK cell exhausting and tumor immune escaping [49]. Immune checkpoints blockade can reduce immune escape of tumor cells and limits tumor growth. Thus, targeting immune checkpoints has become one of the most important ways of immunotherapy [50]. Chemokines have been recognized as small, secreted proteins with important roles in directing the migration of immune cells, which is essential for initiating and providing an effective anti-tumor immune response [51]. Each chemokine can activate several different receptors, which also play a predominant part in the coordination of cell trafficking in the immune response processes [52]. MHC genes are clusters of genes encoding molecules that are primarily associated with innate and adaptive immune responses and other molecular and biological processes [53]. These results elucidated the underlying mechanisms of CYFIP2 in tumor immunity and its influence on immune related genes.

Cellular immunotherapy is a hot field in medicine today. From cancer to systemic lupus erythematosus, cellular immunotherapy has shown promise. With clinical progress, cellular immunotherapy is expected to play an even greater role in the clinic. In this study, we suggested that CYFIP2 might be a promising therapeutic strategy for tumor immunotherapy. However, the immunotherapeutic mechanism of CYFIP2 have not been well elucidated. Shabani et al. made the following points that CYFIP2 participated in the vascular endothelial growth factor receptor signaling pathway and the purine ribonucleoside diphosphate metabolic process, respectively [15]. Moreover, Zhao et al. found that CYFIP2 was closely related to T-cell CD8 + , T-cell CD4 + and neutrophils and was highly associated with tumor mutation burden and microsatellite instability in various cancers [54]. In addition, Tong et al. convinced that CYFIP2 was significantly associated with CD4 + cells, CD8 + cells and a series of immune markers via metabolic pathways and epithelial–mesenchymal transition in clear cell renal cell carcinoma [31]. Our study also indicated that CYFIP2 potentially contributed to regulation of tumor-associated and immune-related pathways and regulated immune-related genes. In a word, possible mechanisms through which CYFIP2 might influence tumor immunity are worthy of in-depth study.

There are several limitations in our study. First, most of the present studies were limited to bioinformatics analysis, further study is required to validate these results. Second, the diagnostic and prognostic value of CYFIP2 were evaluated based on TCGA cohorts, which have not been validated by other datasets or clinical data from real-world. In addition, despite the finding indicating that CYFIP2 expression correlated with both immune cell infiltration and patient survival in cancers, we could not prove that CYFIP2 affects patient survival through immune infiltration.

## Conclusions

Taken together, our present study indicated that CYFIP2 was aberrantly expressed in various tumors and highly involved in tumor-associated and immune-related pathways. The results also indicated that CYFIP2 could be employed as potential diagnostic and prognostic indicator for numerous tumors. In addition, the abnormal expression of CYFIP2 was linked with immune cells infiltration and may affect tumor immunity by acting on immune regulators and immune-related genes, which may provide a foundation for more precise and personalized immunotherapy in future. However, the mechanisms by which CYFIP2 may affect tumor immunity have not been thoroughly investigated by biological experiments. Thus, further experimental and clinical studies in future are required to promote practical application in immunotherapy and prognosis prediction.

## Data Availability

Data are available from the corresponding author upon reasonable request.

## Abbreviations

CYFIP2: Cytoplasmic FMR Interacting Protein 2
LUAD: Lung adenocarcinoma
ESCC: Esophageal squamous cell carcinoma
BC: Bladder carcinoma
KIRC: Kidney renal clear cell carcinoma
KIPP: Kidney renal papillary cell carcinoma
PDAC: Pancreatic ductal adenocarcinoma
LUSC: Lung squamous cell carcinoma
HNSC: Head and neck squamous cell carcinoma
HR: Hazard ratios
CI: Confidence intervals

## References

[1] Barnoud T, Parris JLD, Murphy ME (2019). Common genetic variants in the TP53 pathway and their impact on cancer. J Mol Cell Biol.

[2] Vadapalli S, Abdelhalim H, Zeeshan S, Ahmed Z (2022). Artificial intelligence and machine learning approaches using gene expression and variant data for personalized medicine. Brief Bioinform.

[3] Aveta A, Cilio S, Contieri R, Spena G, Napolitano L, Manfredi C (2023). Urinary MicroRNAs as biomarkers of urological cancers: a systematic review. Int J Mol Sci.

[4] Peng Q, Zhang X, Min M, Zou L, Shen P, Zhu Y (2017). The clinical role of microRNA-21 as a promising biomarker in the diagnosis and prognosis of colorectal cancer: a systematic review and meta-analysis. Oncotarget.

[5] He X, Liu X, Zuo F, Shi H, Jing J (2023). Artificial intelligence-based multi-omics analysis fuels cancer precision medicine. Semin Cancer Biol.

[6] Lin Y, Qian F, Shen L, Chen F, Chen J, Shen B (2019). Computer-aided biomarker discovery for precision medicine: data resources, models and applications. Brief Bioinform.

[7] Inamura K (2018). Clinicopathological characteristics and mutations driving development of early lung adenocarcinoma: tumor initiation and progression. Int J Mol Sci.

[8] Soltis AR, Bateman NW, Liu J, Nguyen T, Franks TJ, Zhang X (2022). Proteogenomic analysis of lung adenocarcinoma reveals tumor heterogeneity, survival determinants, and therapeutically relevant pathways. Cell Rep Med.

[9] Seguin L, Durandy M, Feral CC (2022). Lung adenocarcinoma tumor origin: a guide for personalized medicine. Cancers.

[10] Jackson RS, Cho YJ, Stein S, Liang P (2007). CYFIP2, a direct p53 target, is leptomycin-B sensitive. Cell Cycle.

[11] Manigandan S, Yun JW (2022). Loss of cytoplasmic FMR1-interacting protein 2 (CYFIP2) induces browning in 3T3-L1 adipocytes via repression of GABA-BR and activation of mTORC1. J Cell Biochem.

[12] Biembengut IV, Silva ILZ, Souza T, Shigunov P (2021). Cytoplasmic FMR1 interacting protein (CYFIP) family members and their function in neural development and disorders. Mol Biol Rep.

[13] Li Y, Song X, Liu L, Yue L (2021). NUAK2 silencing inhibits the proliferation, migration and epithelial-to-mesenchymal transition of cervical cancer cells via upregulating CYFIP2. Mol Med Rep.

[14] Lin J, Liao S, Li E, Liu Z, Zheng R, Wu X (2020). circCYFIP2 acts as a sponge of miR-1205 and affects the expression of its target gene E2F1 to regulate gastric cancer metastasis. Mol Ther Nucleic Acids.

[15] Shabani S, Khayer N, Motalebzadeh J, Majidi Zadeh T, Mahjoubi F (2021). Characterization of pathways involved in colorectal cancer using real-time RT-PCR gene expression data. Gastroenterol Hepatol Bed Bench.

[16] Consortium GT (2013). The genotype-tissue expression (GTEx) project. Nat Genet.

[17] Ponten F, Schwenk JM, Asplund A, Edqvist PH (2011). The human protein atlas as a proteomic resource for biomarker discovery. J Intern Med.

[18] Li T, Fu J, Zeng Z, Cohen D, Li J, Chen Q (2020). TIMER2.0 for analysis of tumor-infiltrating immune cells. Nucleic Acids Res.

[19] Hutter C, Zenklusen JC (2018). The cancer genome atlas: creating lasting value beyond its data. Cell.

[20] Lanczky A, Gyorffy B (2021). Web-Based survival analysis tool tailored for medical research (KMplot): development and implementation. J Med Internet Res.

[21] Brunner M, Mullen L, Jauk F, Oliver J, Cayol F, Minata J (2022). Automatic integration of clinical and genetic data using cBioPortal. Stud Health Technol Inform.

[22] Chandrashekar DS, Karthikeyan SK, Korla PK, Patel H, Shovon AR, Athar M (2022). UALCAN: An update to the integrated cancer data analysis platform. Neoplasia.

[23] Vasaikar SV, Straub P, Wang J, Zhang B (2018). LinkedOmics: analyzing multi-omics data within and across 32 cancer types. Nucleic Acids Res.

[24] Szklarczyk D, Kirsch R, Koutrouli M, Nastou K, Mehryary F, Hachilif R (2023). The STRING database in 2023: protein-protein association networks and functional enrichment analyses for any sequenced genome of interest. Nucleic Acids Res.

[25] The Gene Ontology C (2019). The gene ontology resource: 20 years and still GOing strong. Nucleic Acids Res.

[26] Kanehisa M, Furumichi M, Sato Y, Ishiguro-Watanabe M, Tanabe M (2021). KEGG: integrating viruses and cellular organisms. Nucleic Acids Res.

[27] Ru B, Wong CN, Tong Y, Zhong JY, Zhong SSW, Wu WC (2019). TISIDB: an integrated repository portal for tumor-immune system interactions. Bioinformatics.

[28] Goenka A, Khan F, Verma B, Sinha P, Dmello CC, Jogalekar MP (2023). Tumor microenvironment signaling and therapeutics in cancer progression. Cancer Commun.

[29] Jiao S, Li N, Cai S, Guo H, Wen Y (2017). Inhibition of CYFIP2 promotes gastric cancer cell proliferation and chemoresistance to 5-fluorouracil through activation of the Akt signaling pathway. Oncol Lett.

[30] Chang YS, Huang HD, Yeh KT, Chang JG (2017). Identification of novel mutations in endometrial cancer patients by whole-exome sequencing. Int J Oncol.

[31] Tong J, Meng X, Lv Q, Yuan H, Li W, Xiao W (2021). The downregulation of prognosis- and immune infiltration-related gene CYFIP2 serves as a novel target in ccRCC. Int J Gen Med.

[32] Fois SS, Paliogiannis P, Zinellu A, Fois AG, Cossu A, Palmieri G (2021). Molecular epidemiology of the main druggable genetic alterations in non-small cell lung cancer. Int J Mol Sci.

[33] Castellanos E, Feld E, Horn L (2017). Driven by mutations: the predictive value of mutation subtype in EGFR-Mutated non-small cell lung cancer. J Thorac Oncol.

[34] Nishiyama A, Nakanishi M (2021). Navigating the DNA methylation landscape of cancer. Trends Genet.

[35] Oliver J, Garcia-Aranda M, Chaves P, Alba E, Cobo-Dols M, Onieva JL (2022). Emerging noninvasive methylation biomarkers of cancer prognosis and drug response prediction. Semin Cancer Biol.

[36] Ehrlich M (2019). DNA hypermethylation in disease: mechanisms and clinical relevance. Epigenetics.

[37] Cao X, Geng Q, Fan D, Wang Q, Wang X, Zhang M (2023). m(6)A methylation: a process reshaping the tumour immune microenvironment and regulating immune evasion. Mol Cancer.

[38] Punekar SR, Velcheti V, Neel BG, Wong KK (2022). The current state of the art and future trends in RAS-targeted cancer therapies. Nat Rev Clin Oncol.

[39] Zhao Y, Guo S, Deng J, Shen J, Du F, Wu X (2022). VEGF/VEGFR-Targeted therapy and immunotherapy in non-small cell lung cancer: targeting the tumor microenvironment. Int J Biol Sci.

[40] Wang Q, Zeng A, Zhu M, Song L (2023). Dual inhibition of EGFR-VEGF: An effective approach to the treatment of advanced non-small cell lung cancer with EGFR mutation (Review). Int J Oncol.

[41] Sanaei MJ, Razi S, Pourbagheri-Sigaroodi A, Bashash D (2022). The PI3K/Akt/mTOR pathway in lung cancer; oncogenic alterations, therapeutic opportunities, challenges, and a glance at the application of nanoparticles. Transl Oncol.

[42] Quan Z, Yang Y, Zheng H, Zhan Y, Luo J, Ning Y (2022). Clinical implications of the interaction between PD-1/PD-L1 and PI3K/AKT/mTOR pathway in progression and treatment of non-small cell lung cancer. J Cancer.

[43] Gupta M, Chandan K, Sarwat M (2022). Natural products and their derivatives as immune check point inhibitors: targeting cytokine/chemokine signalling in cancer. Semin Cancer Biol.

[44] Lin A, Fang J, Cheng Q, Liu Z, Luo P, Zhang J (2022). B Cell receptor signaling pathway mutation as prognosis predictor of immune checkpoint inhibitors in lung adenocarcinoma by bioinformatic analysis. J Inflamm Res.

[45] Morton LT, Wachsmann TLA, Meeuwsen MH, Wouters AK, Remst DFG, van Loenen MM (2022). T cell receptor engineering of primary NK cells to therapeutically target tumors and tumor immune evasion. J Immunother Cancer.

[46] de Visser KE, Joyce JA (2023). The evolving tumor microenvironment: from cancer initiation to metastatic outgrowth. Cancer Cell.

[47] Arner EN, Rathmell JC (2023). Metabolic programming and immune suppression in the tumor microenvironment. Cancer Cell.

[48] Li HX, Wang SQ, Lian ZX, Deng SL, Yu K (2022). Relationship between tumor infiltrating immune cells and tumor metastasis and its prognostic value in cancer. Cells.

[49] Guo Z, Zhang R, Yang AG, Zheng G (2023). Diversity of immune checkpoints in cancer immunotherapy. Front Immunol.

[50] Ghaedrahmati F, Esmaeil N, Abbaspour M (2023). Targeting immune checkpoints: how to use natural killer cells for fighting against solid tumors. Cancer Commun (Lond).

[51] Ozga AJ, Chow MT, Luster AD (2021). Chemokines and the immune response to cancer. Immunity.

[52] Griffith JW, Sokol CL, Luster AD (2014). Chemokines and chemokine receptors: positioning cells for host defense and immunity. Annu Rev Immunol.

[53] Sachini N, Papamatheakis J (2017). NF-Y and the immune response: dissecting the complex regulation of MHC genes. Biochim Biophys Acta Gene Regul Mech.

[54] Zhao Z, He S, Yu X, Lai X, Tang S, Mariya ME (2022). Analysis and experimental validation of rheumatoid arthritis innate immunity gene CYFIP2 and Pan-Cancer. Front Immunol.

